# Geospatial analysis of emergency department visits for targeting community-based responses to the opioid epidemic

**DOI:** 10.1371/journal.pone.0175115

**Published:** 2017-03-31

**Authors:** Daniel A. Dworkis, Lauren A. Taylor, David A. Peak, Benjamin Bearnot

**Affiliations:** 1 Harvard Medical School, Department of Emergency Medicine, Boston, Massachusetts, United States of America; 2 Massachusetts General Hospital, Department of Emergency Medicine, Boston, Massachusetts, United States of America; 3 Harvard Management Business School, Boston, Massachusetts, United States of America; 4 Harvard Medical School, Department of Medicine, Boston, Massachusetts, United States of America; 5 Massachusetts General Hospital, Division of General Internal Medicine, Department of Medicine, Boston, Massachusetts, United States of America; Columbia University, UNITED STATES

## Abstract

The opioid epidemic in the United States carries significant morbidity and mortality and requires a coordinated response among emergency providers, outpatient providers, public health departments, and communities. Anecdotally, providers across the spectrum of care at Massachusetts General Hospital (MGH) in Boston, MA have noticed that Charlestown, a community in northeast Boston, has been particularly impacted by the opioid epidemic and needs both emergency and longer-term resources. We hypothesized that geospatial analysis of the home addresses of patients presenting to the MGH emergency department (ED) with opioid-related emergencies might identify “hot spots” of opioid-related healthcare needs within Charlestown that could then be targeted for further investigation and resource deployment. Here, we present a geospatial analysis at the United States census tract level of the home addresses of all patients who presented to the MGH ED for opioid-related emergency visits between 7/1/2012 and 6/30/2015, including 191 visits from 100 addresses in Charlestown, MA. Among the six census tracts that comprise Charlestown, we find a 9.5-fold difference in opioid-related ED visits, with 45% of all opioid-related visits from Charlestown originating in tract 040401. The signal from this census tract remains strong after adjusting for population differences between census tracts, and while this tract is one of the higher utilizing census tracts in Charlestown of the MGH ED for all cause visits, it also has a 2.9-fold higher rate of opioid-related visits than the remainder of Charlestown. Identifying this hot spot of opioid-related emergency needs within Charlestown may help re-distribute existing resources efficiently, empower community and ED-based physicians to advocate for their patients, and serve as a catalyst for partnerships between MGH and local community groups. More broadly, this analysis demonstrates that EDs can use geospatial analysis to address the emergency and longer-term health needs of the communities they are designed to serve.

## Introduction

The United States is facing an opioid epidemic defined by historically high rates of opioid associated overdoses and deaths. More than 2.4 million Americans have a severe opioid-use disorder (OUD) and mortality from opioid use is now the leading cause of accidental death in the United States [1, 2]. In 2014, the highest rate of opioid-related ED visits was in Massachusetts (441.6 visits per 100,000 population), which was more than 14 times higher than the lowest rate in Iowa (31.1) [3]. In 2015, the last year for which data are available, 1,659 Massachusetts residents are estimated to have lost their lives to opioid overdose, representing an all-time high based on available records [4]. At least 75% of the states’ 351 municipalities have experienced an opioid related death since 2014 [5, 6]. Massachusetts policymakers and practitioners are attempting to devise effective strategies to limit the health impact of opioid addiction [7].

Charlestown is a community of approximately 16.5 thousand people in the northeast of the city, which is perennially described as a particularly high-risk community with regards to opioids [7–10]. Within Charlestown there are six census tracts with 2010 population levels ranging from 1,606 to 3,900. The MGH Charlestown Community Healthcare Center is a multidisciplinary community health center where many Charlestown residents get their primary and addiction care. Opioid addiction outreach efforts are currently limited to events held in the health center due to lack of knowledge about where those with substance use disorder live in the community. Furthermore, resources for addressing opioid-related healthcare needs in the community are limited and the most efficient way to distribute these resources within Charlestown is unclear.

Here, we present a geospatial analysis of the home addresses of patients from Charlestown presenting to the Massachusetts General Hospital (MGH) emergency department (ED) with opioid-related emergencies and identify a hotspot at census tract 040401. This analysis was motivated by an interest in utilizing ED visit data to identify “hot spots” of high opiod-related healthcare utilization within Charlestown to target further investigation, resource deployment, and community-based responses to change the course of the opioid overdose epidemic. Furthermore, while a single site analysis by definition does not consider visits to other EDs or health centers, it does highlight community level risk as viewed from the common model of the dyad of a “mothership” hospital and a “satellite” health center embedded in that community.

## Materials and methods

We performed a geospatial analysis of home address locations based on spatially aggregated patient record data extracted from MGH ED records for all visits to the MGH ED between 2012 and 2015. Self-reported home address, chief complaint, diagnosis on discharge from the emergency department, diagnosis on discharge from the hospital, and basic demographics were collected for all patients who presented to the MGH ED between July 1, 2012 and June 30, 2015. International Statistical Classification of Diseases and Related Health Problem codes were not available in this dataset, so text-based discharge diagnoses were used. If a patient was discharged from the ED, the recorded hospital discharge diagnosis would be the same as the ED discharge diagnosis; if the patient was admitted to the hospital, the hospital discharge diagnosis reflected the diagnosis on discharge from the hospital which may or may not be the same as the ED discharge diagnosis.

In July 2014, the MGH ED changed electronic medical record (EMR) providers and diagnosis upon discharge from the ED was no longer recorded distinctly from hospital discharge diagnosis during the remainder of the study period (July 11, 2014-June 30, 2015). Where available before the EMR transition, both ED and hospital discharge diagnoses were examined for risk of opioid overdose, as explained below. After the EMR transition, only hospital discharge diagnosis was considered as this was all the available data. All data manipulation and statistical analyses were developed and executed using the R programming language [11]. This study was approved by the institutional review board at Partners Healthcare.

Opioid-related ED visits were identified using a text search of chief complaint and discharge diagnosis selecting for terms involving narcotic overdose or drug abuse, or involving the name of a specific narcotic medication or naloxone. Specific terms searched for were: "HEROIN", "OVERDOSE", "OPIOID", "OPIATE", "METHADONE", "OPIUM", "NARCOTIC", "NARCAN", "NALOXONE", "FENTANYL", "OXYCODONE", "OXYCONTIN", "PERCOCET", "MORPHINE", "MORPHONE", "DILAUDID", "HYDROMORPHONE", "MS CONTIN", "HYDROCODONE", "CODINE", "TRAMADOL", "DRUG ABUSE", “DRUG”, and “VICODIN”.. Since the chief complaint for each visit was entered as free-text, partial string matching executed using the “grepl” function in R was employed to account for lexical variability. To improve the specificity of this approach, we also searched for matches to the terms “DRUG RASH", "EXPERIMENTAL DRUG", "DRUG RX", "DRUG REACTION", and "DRUGGED" which were structurally similar to the terms of interest but which were thought unlikely to represent opioid-related ED visits and visits with chief complaints or discharge diagnoses matching these second set of terms were excluded from the analysis.

Addresses in MA were geocoded using the United States Census Geocoder using address batch geography lookups matched to US Census 2010 data vintage [12]. To de-identify individual patient addresses, we spatially aggregated home addresses at the US census tract level at this step and only considered spatially aggregated data as opposed to exact addresses in further analyses. Census tracts were chosen over zip codes for two reasons: first, census tracts are spatially stable over 10 year periods between US census administrations, unlike zip codes which might change boundaries frequently. Second, census tracts generally offer a higher degree of spatial granularity than zip codes; Charlestown is divided into six census tracts but has only one zip code. Geospatial analysis was performed using QGIS, with base maps obtained from Massachusetts Office of Geographic Information (MASSGIS)[13, 14]. Choropleths for hotspot analysis were built in QGIS using Jenks Natural Breaks optimization. For population adjustment, theoretical population-weighted distributions were obtained by distributing the total number of observed visits or addresses across Charlestown census tracts according to their 2010 population from the US Census 2010 data, which was embedded in the MASSGIS data.

## Results

Between July 1, 2012 and June 30, 2015, there were 313,085 patient visits to the MGH ED from 151,250 distinct addresses, including 286,555 patient visits from 132,253 addresses in Massachusetts. From Charlestown, there were 10,577 visits during the study period from 2,822 addresses. Among these 10,577 visits, we identified 210 visits from 111 addresses as opioid-related ED visits; 9,777 visits (92.4%) including 191 opioid-related visits (91.0%) were successfully geocoded. There was no significant association between ability to geocode a visit from Charlestown and whether the visit was opioid-related or not (Chi-squared test, p-value = 0.49). These 191 geocoded, opioid-related visits from 100 addresses in Charlestown were the basis for further geospatial analysis described below.

**Fig 1:**
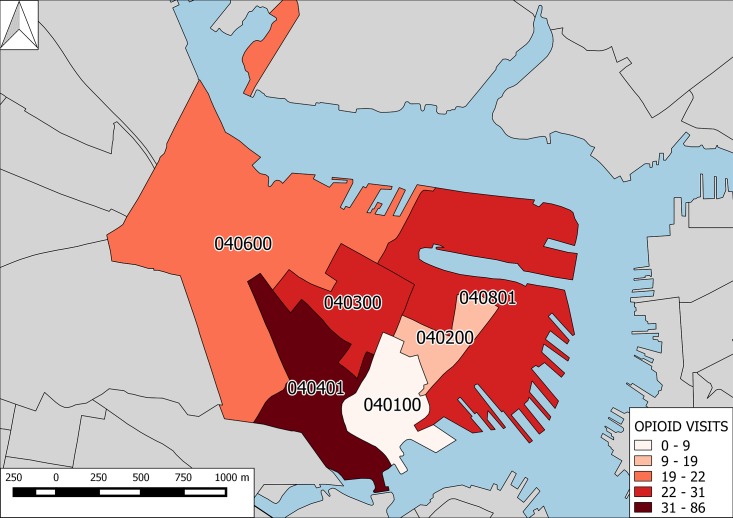
Opioid-related ED visits from Charlestown, MA. Choropleth map of Charlestown, MA at 1:17,000 scale with census tracts colored by level of opioid-related ED visits to MGH. Grey areas show boundaries of census tracts outside of Charlestown. Compass arrow at upper left points north. Blue shaded areas represent water and show the confluence of the Massachusetts Bay and the heads of the Charles, Mystic, and Chelsea Rivers.

Census-tract level analysis showed obvious heterogeneity and a 9.5-fold variability in opioid-related ED visits per census tract with the 86 opioid-related ED visits (45% of all opioid-related visits from Charlestown) originating from addresses in census-tract 040401 compared to only 9 (0.5%) opioid-related ED visits from census-tract 040100. Fig 1 shows a geospatial analysis of opioid-related ED visits at the census-tract level highlighting both the “hot spot” at census tract 040401 and low-levels of opioid-related ED visits in bordering tract 040100.

There was also a large variability in the number of visits from each address within a census tract, with between 1 and 21 opioid related ED visits per address. S1 Fig shows the breakdown of opioid-related visits per address across Charlestown. To address potential confounding in the spatial signal by address with high numbers of visits per address, we examined the spatial distribution of addresses with opioid-related ED visits and find a five-fold variability with a pattern similar to that for opioid-related visits: there are 35 addresses with opioid-related visits in census tract 040401 (35% of all addresses in Charlestown with opioid-related visits) compared to only 7 addresses in census tract 040100 (7%).

**Fig 2:**
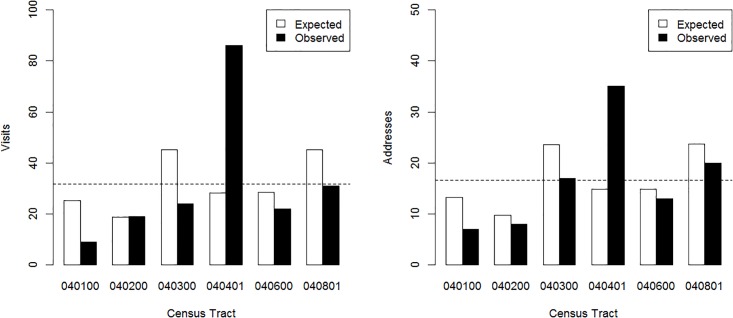
Opioid-related ED visits and addresses compared to population levels. Bar graph of observed vs population-weighted expected opioid-related visits (Left), and addresses (Right) from each census tract in Charlestown, MA. Expected distributions were calculated by weighting the total number of opioid-related ED visits (Left) or addresses (Right) from Charlestown by the population in each census tract. Observed distributions are the actual number of opioid-related ED visits (Left) or addresses (Right) from each census tract. The horizontal dotted line represents a simple (i.e. not population-weighted) average obtained by distributing the total number of opioid-related ED visits (Left) or addresses (Right) equally across all census tracts.

To account for the known variability in population across census tracts in Charlestown, we compared the observed distributions of opioid-related ED visits and addresses with opioid-related ED visits to theoretical expected distributions based on population weighting. Fig 2 compares the observed and expected distributions for visits (left) and addresses (right), suggesting that the “hot spot” at 040401 is not simply an artifact of population differences between census tracts.

**Fig 3:**
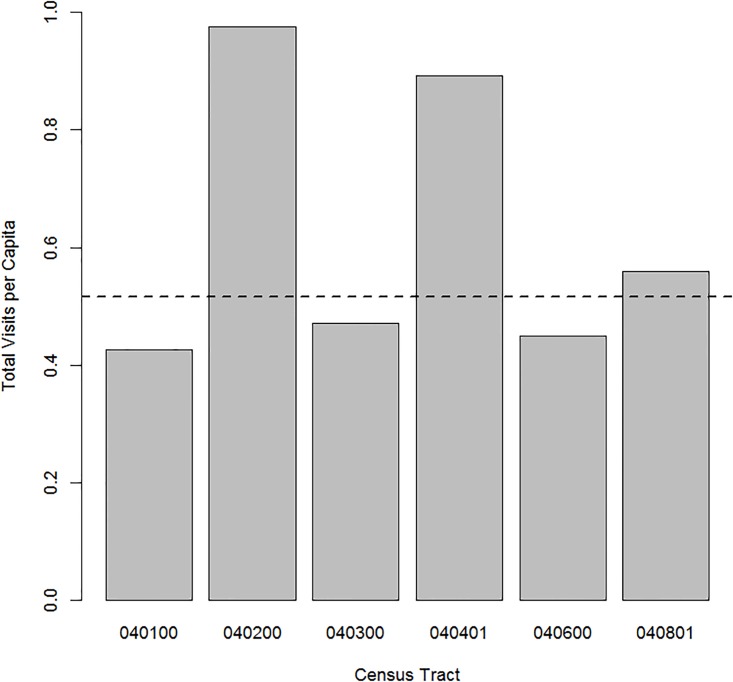
Emergency department visits per capita. Bar graph of all visits (opioid-related and otherwise) to the MGH ED per capita from each census tract in Charlestown, MA based on the census tract level population from the 2010 US Census. The dashed line represents the median per capita visits across all census tracts in Charlestown (0.55).

**Fig 4:**
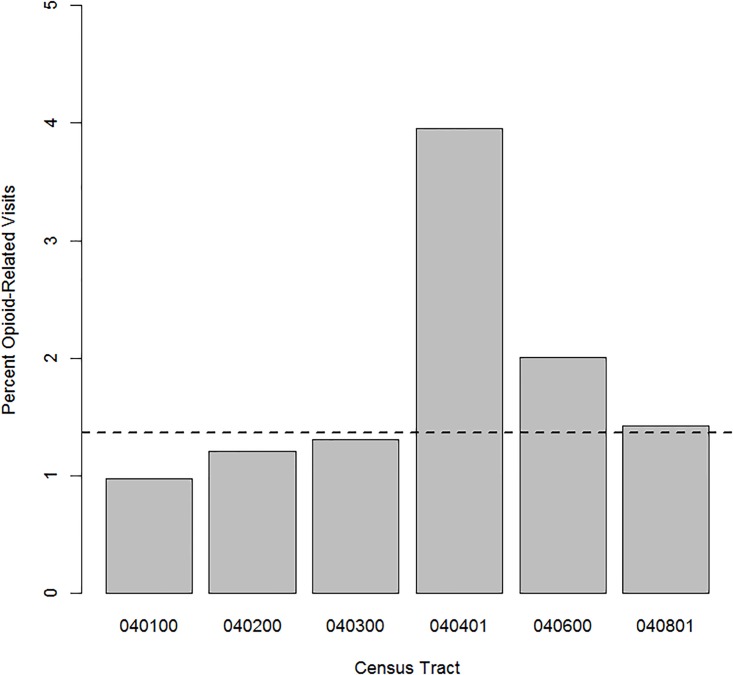
Percent of all visits which are opioid-related. Bar graph of the percent of all visits to the MGH ED which are opioid-related from each census tract in Charlestown, MA. The dashed line represents the median percent of visits which are opioid-related from all census tracts within Charlestown (1.4%).

Since people living in different areas may utilize emergency health resources at different rates, we looked at census-tract level differences in any-cause visit rates to the MGH ED. Fig 3 compares per capita visit rates between census tracts and shows that census tract 040401 is a relatively high-utilizer area of Charlestown with 0.89 visits per capita compared to a median across Charlestown of 0.51 visits per capita and a maximum of 0.98 visits per capita in census tract 040200. To consider the possibility that the opioid-related hot spot at census tract 040401 is simply related to high-utilization by this census tract in general, we then examined the percent of all visits from each census tract which were opioid-related. Fig 4 displays this distribution across Charlestown, demonstrating that approximately 4.0% of all visits from census tract 040401 are opioid-related, compared to a median of 1.4% and 1.2% in census tract 040200. Taken together, Figs 3 and 4 suggest that the high level of opioid-related visits from census tract 040401 is not simply a reflection of high ED use rates in general.

## Discussion

Our study characterizes geographic variations in opioid-related ED visits among census tracts in Charlestown, Massachusetts. The wide heterogeneity observed was not driven by overall ED usage rates or population size differences between census tracts. These results emphasize that while geographically small, Charlestown is a community exhibiting significant spatial heterogeneity in opioid-related ED utilization with substructure showing a spatial hot spot at census tract 040401. Within Charlestown, these data can be used to facilitate both further investigation into factors effecting opioid use disorder and opioid-related healthcare utilization, as well as efficient distribution of local resources addressing the opioid epidemic. These may include emergency response resources such as bystander naloxone as well as longer term responses such as addiction treatment centers. Additionally, the underlying reason why census tract 040401 is a hotspot of opioid-related ED visits is unclear, and further work is needed to better explore and understand the medical, social, and structural factors in this area.

In a broader sense, this study demonstrates the potential value of emergency department data in informing health policy interventions in response to the opioid epidemic and other public health crises. Continuing to collect, and appropriately analyze, emergency visit data and other data that can be geospatially analyzed may serve as a key tool in understanding the potential for place-based patterns of health outcomes and health care utilization [15]. In instances where conditions are strongly tied to place-based social determinants of health, granular data like home addresses and other demographics can be used to target limited resources where the problem is most severe.

Furthermore, this analysis highlights how EDs are uniquely positioned within health systems to provide critical intelligence to other parts of the healthcare system, particularly health clinics and other outpatient facilities, about the risks community facilities may wish to address [16]. This intelligence could also prove useful in the creation of more effective community benefit spending strategies that leverage ED data to prioritize investment in community-based organizations located in particular neighborhoods or addressing specific health challenges.

## Limitations

In this study, we examined the utility of geospatial analysis of opioid-related ED visits from an a prioi selected community to the ED of a single quaternary care center. Our dataset therefore excludes ED visits to other nearby hospitals, health centers, and urgent cares, so our results should be interpreted more as exploring how an ED can interface with a specific community to guide resource distribution than as providing all-encompassing information about Charlestown specifically. Additionally, people do not necessarily use opioids where they live, so targeting resources to census tracts identified by home address will likely only address part of the opioid-related risk a community carries. Future work should incorporate ED visits to other nearby emergency departments as well as pre-hospital data on emergency medical response to opioid overdoses to further characterize community level distribution of risk.

## Supporting information

S1 Fig Opioid-related ED visits per address. Stripchart of the number of opioid-related ED visits per address across each census tract in Charlestown, MA. Each circle represents a distinct address within a given census tract. A spatial jitter has been applied to facilitate viewing degenerate points.
(TIF)

## References

[1] Substance Abuse and Mental Health Services Administration, Center for Behavioral Health Statistics and Quality. Behavioral health trends in the United States: Results from the 2014 National Survey on Drug Use and Health. Rockville (MD): Substance Abuse and Mental Health Services Administration; 2015 37 p. http://www.samhsa.gov/data/sites/default/files/NSDUH-FRR1-2014/NSDUH-FRR1-2014.pdf.

[2] Rudd RA, Aleshire N, Zibbell JE, Matthew Gladden R. Increases in drug and opioid overdose deaths—United States, 2000–2014. Morbidity and Mortality Weekly Report. 2016 1 1; 64(50);1378–82. 26720857 10.15585/mmwr.mm6450a3

[3] Weiss AJ, Elixhauser A, Barrett ML, Steiner CA, Bailey MK, O'Malley L. Opioid-Related Inpatient Stays and Emergency Department Visits by State, 2009–2014 HCUP Statistical Brief #219. 12 2016 Agency for Healthcare Research and Quality, Rockville, MD http://www.hcup-us.ahrq.gov/reports/statbriefs/sb219-Opioid-Hospital-Stays-ED-Visits-by-State.pdf.

[4] Massachusetts Department of Health and Human Services, Department of Public Health. Data Brief: Opioid-related Overdose Deaths Among Massachusetts Residents. Boston (MA): Massachusetts Department of Public Health; 2016 3 p. http://www.mass.gov/eohhs/docs/dph/quality/drugcontrol/county-level-pmp/opioid-related-overdose-deaths-among-ma-residents-august-2016.pdf

[5] Massachusetts Department of Health and Human Services, Department of Public Health. Overdose Deaths by City/Town. Boston (MA): Massachusetts Department of Public Health; 2016 10 p. http://www.mass.gov/eohhs/docs/dph/quality/drugcontrol/county-level-pmp/overdose-deaths-by-city-town-august-2016.pdf

[6] Massachusetts Department of Health and Human Services. Massachusetts Opioid Epidemic: A data visualization of findings from the Chapter 55 report. Boston (MA) Massachusetts Department of Health and Human Services; 2016 http://www.mass.gov/chapter55/

[7] Massachusetts Governor’s Opioid Working Group. Recommendations of the Governor's Opioid Working Group. Boston (MA): Governor’s Opioid Working Group; 2015 47 p. http://www.mass.gov/eohhs/gov/departments/dph/stop-addiction/recommendations-from-the-governors-opioid-addiction-working-group.html

[8] Center for Community Health Improvement, Massachusetts General Hospital. 2016 Community Health Needs Assessment: Adolescent Substance Use and Mental Health. Boston (MA): Massachusetts General Hospital; 2016: 30 p. http://www.massgeneral.org/cchi/assets/pdf/MGH%20CHNA%202016_Final_ForWeb.pdf

[9] MacQuarrie B. As Charlestown opioid crisis deepens, a new clinic opens. Boston Globe. 2015 Dec 14.

[10] Bearnot B, Eisenberg M. STAT News. It’s time to call the opioid epidemic a public health emergency. 2015 Sep 22. https://www.statnews.com/2016/09/22/opioid-epidemic-public-health-emergency/

[11] R Core Team. R: A language and environment for statistical computing. R Foundation for Statistical Computing, Vienna, Austria; 2015 https://www.R-project.org/.

[12] United States Census Geocoder; 2016. http://geocoding.geo.census.gov

[13] QGIS Development Team. QGIS Geographic Information System. Open Source Geospatial Foundation Project; 2016. http://www.qgis.org/

[14] Massachusetts Office of Geographic Information (MassGIS); 2016. http://www.mass.gov/anf/research-and-tech/it-serv-and-support/application-serv/office-of-geographic-information-massgis/

[15] Lee DC, Doran KM, Polsky D, Cordova E, Carr BG. Geographic variation in the demand for emergency care: A local population-level analysis. Healthcare. 2015; 4(2): 98–103. doi: 10.1016/j.hjdsi.2015.05.003 27343158 10.1016/j.hjdsi.2015.05.003

[16] Dworkis DA, Peak DA, Ahn J, Joseph TA. Reaching Out of the Box: Effective Emergency Care Requires Looking Outside the Emergency Department. Western Journal of Emergency Medicine. 2016 7;17(4):484–486. doi: 10.5811/westjem.2016.5.30244 27429705 10.5811/westjem.2016.5.30244 PMC4944811

