# Supplementary figures and images for: Geospatial analysis of emergency department visits for targeting community-based responses to the opioid epidemic
Source: PLoS One. 2017 Mar 31;12(3):e0175115. doi: 10.1371/journal.pone.0175115 (PMC5376332; doi:10.1371/journal.pone.0175115)

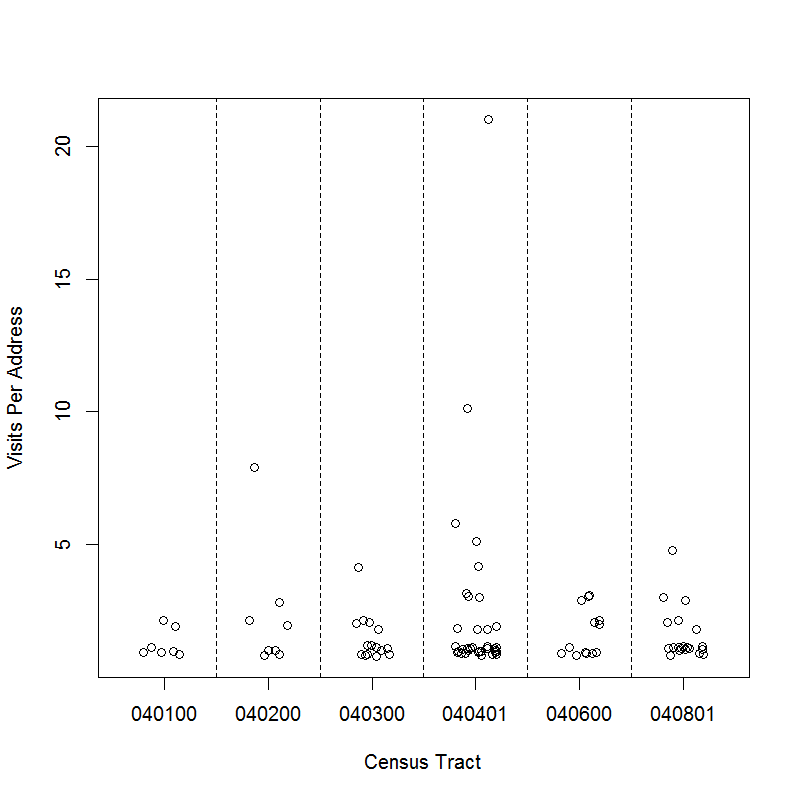

Supplement: S1 Fig — Stripchart of the number of opioid-related ED visits per address across each census tract in Charlestown, MA. Each circle represents a distinct address within a given census tract. A spatial jitter has been applied to facilitate viewing degenerate points. (TIF) [file pone.0175115.s001.tif]
